# Integration of Diffusion Transformer and Knowledge Graph for Efficient Cucumber Disease Detection in Agriculture

**DOI:** 10.3390/plants13172435

**Published:** 2024-08-31

**Authors:** Ruiheng Li, Xiaotong Su, Hang Zhang, Xiyan Zhang, Yifan Yao, Shutian Zhou, Bohan Zhang, Muyang Ye, Chunli Lv

**Affiliations:** China Agricultural University, Beijing 100083, China

**Keywords:** cucumber disease detection, diffusion transformer, deep learning in agriculture, knowledge graph integration, precision agriculture technologies

## Abstract

In this study, a deep learning method combining knowledge graph and diffusion Transformer has been proposed for cucumber disease detection. By incorporating the diffusion attention mechanism and diffusion loss function, the research aims to enhance the model’s ability to recognize complex agricultural disease features and to address the issue of sample imbalance efficiently. Experimental results demonstrate that the proposed method outperforms existing deep learning models in cucumber disease detection tasks. Specifically, the method achieved a precision of 93%, a recall of 89%, an accuracy of 92%, and a mean average precision (mAP) of 91%, with a frame rate of 57 frames per second (FPS). Additionally, the study successfully implemented model lightweighting, enabling effective operation on mobile devices, which supports rapid on-site diagnosis of cucumber diseases. The research not only optimizes the performance of cucumber disease detection, but also opens new possibilities for the application of deep learning in the field of agricultural disease detection.

## 1. Introduction

In agricultural production, the timely and accurate detection and diagnosis of crop diseases are crucial for enhancing yield and quality [1,2,3]. Particularly for widely cultivated vegetables such as cucumbers, early detection and management of diseases are directly linked to the economic benefits of agriculture [4,5]. Traditional methods of disease detection often rely on manual inspection [6], which is time-consuming, labor-intensive, and highly subjective [7,8]. Hence, there is an increasing demand for automated systems capable of accurately and efficiently detecting cucumber diseases.

With the rapid development of information technology and artificial intelligence, the use of advanced computational models for disease detection has become a research focus [9,10]. It was analyzed by Ben Ayed et al. [11] that by 2050, the world population is expected to exceed 9 billion, necessitating a 70% increase in agricultural and food production to meet the demand, posing a severe challenge for the agri-food industry, thereby underlining the urgent need to address plant disease issues; in response, Poornappriya TS et al. [12] utilized machine learning and image processing techniques to handle disease detection and classification issues in rice, designing a system to identify diseases and provide information on pesticide choices. Moreover, Sivalingam Vidya et al. [13] employed CNN for preprocessing and feature extraction, and used an RNN-based Long Short-Term Memory (LSTM) method for further feature extraction and classification. Experimental results show that the LSTM method achieved the highest recognition accuracy compared to other methods; Krishna Rajashree et al. [14] constructed a model based on weather parameters and RNN to predict the incidence of nut fruit rot, effectively reducing prediction inaccuracies.

Wang et al. [15] addressed the challenges of detecting and classifying plant leaf diseases by proposing a novel method called Deep Block Attention SSD, designed for the identification and classification of plant leaf diseases; Qadri, Syed Asif Ahmad et al. [16] introduced a deep learning solution for plant leaf disease detection and segmentation using the YOLOv8 model. Experimental results demonstrate this model’s strong performance in accurately detecting and segmenting diseased areas; building on this, He et al. [17] developed a method for detecting cotton pests and diseases in complex natural environments using an improved YOLOv9 model. The results indicate that this model achieved an accuracy rate of 93%, which is higher than that of earlier models such as YOLOv7 and YOLOv8x; Wu et al. [18] proposed the Disease Segmentation Detection Transformer based on several enhancements to DETR, for effectively segmenting leaf disease spots. Experimental results show that our proposed model achieves segmentation outcomes with a disease grading accuracy of 0.9640.

However, existing disease detection methods often overlook the rich professional knowledge accumulated in the agricultural field, failing to effectively integrate traditional knowledge with modern technology [19]. Current disease detection technologies are mostly technology-driven, for example TinySegformer [20] follows a pattern of technology-first, application-second, which often leads to technology development detached from the specific needs of actual agricultural production. Memon Muhammad Suleman et al. [21] proposed a new strategy to enhance the effectiveness of deep learning models—the Meta Deep Learning model—and implemented a dataset for cotton crops. This dataset was trained on various models, including custom CNN, VGG16, ResNet50, and Meta Deep Learning models. Experimental results indicate that the proposed models perform exceptionally well on the cotton dataset, with an accuracy of 98.53%. However, these models still face limitations in processing low-resolution images and reducing model size, making it difficult to deploy on mobile devices; to address this, Chukkapalli Sai Sree Laya et al. [22] developed an interconnected cooperative ecosystem, defining sensors and their communications between different entities along with a cloud-supported cooperative hub. Ontologies for member farms and cooperatives were developed to capture shared resources stored in the cloud, data between member farms and cooperatives, and various interactions. These can then assist in generating AI-supported insights for farmers and cooperatives. Several cooperative agricultural use-case scenarios have been discussed to demonstrate the capabilities of the smart cooperative ecosystem.

Verónica Saiz-Rubio et al. [23] demonstrated how to make decisions in modern data-based agriculture that are sustainable and profitable while reducing environmental harm. Experimental analysis confirmed that agricultural management systems can handle farm data to coordinate outcomes for customized solutions at each farm. This aid provided to farmers in the form of digital solutions combines robotics with artificial intelligence, introducing the forthcoming concept of Agriculture 5.0; Ismail Nazrul et al. [24] proposed an efficient machine vision system based on the most advanced deep learning technologies and a stacked ensemble method, achieving an average accuracy of 99.2% for apples and 98.6% for bananas, but it is entirely based on the appearance of the fruits and only utilizes a single view of the fruit images, lacking a multi-view vision system training.

This study aims to enhance the accuracy and efficiency of cucumber disease detection by integrating a knowledge graph with a diffusion Transformer-based deep learning model. The specific research objectives include: Constructing an agricultural knowledge graph that covers types of cucumber diseases, conditions of occurrence, and typical symptoms; Developing a diffusion Transformer model integrated with the knowledge graph, utilizing diffusion attention mechanisms and diffusion loss functions to optimize the accuracy of disease recognition and classification; Implementing lightweight deployment of the model on mobile platforms to support rapid on-site disease diagnosis. Through these objectives, we hope to advance the development of smart agricultural technology, particularly in the application of disease management and precision agriculture practices. The innovations of this paper are as follows:

1. Based on the agricultural knowledge graph-diffusion Transformer: An agricultural knowledge graph was constructed, systematically integrating relevant information on cucumber diseases, including types of diseases, conditions for occurrence, and typical symptoms. Based on this knowledge graph, a diffusion Transformer model was developed, guiding the model to more accurately identify and classify cucumber diseases through the knowledge in the graph.
2. Diffusion attention mechanism: In the Transformer architecture, the diffusion attention mechanism was innovatively introduced. This mechanism effectively processes information from different data sources, such as image data and the knowledge graph, enhancing the model’s focus on key features, thereby improving recognition precision and robustness.
3. Diffusion loss function: To optimize the training process and enhance the model’s generalization ability, a diffusion loss function was designed. This novel loss function considers the detection difficulty of different disease categories, adaptively adjusting the loss weights, effectively alleviating the issue of class imbalance.
4. Mobile platform cucumber disease detection system: Addressing the application needs in agricultural fields, the aforementioned model was deployed on a mobile platform. By optimizing algorithms and model structure, the system’s operational efficiency and real-time performance on mobile devices are ensured, enabling farmers to conduct disease detection directly in the fields using smartphones or portable devices.

These innovations not only enhance the accuracy of cucumber disease detection, but also provide new research directions and practical applications for disease detection technologies for other crops. Future work will further explore the expansion and deepening of the knowledge graph and the application potential of the diffusion model in broader agricultural data processing.

## 2. Related Work

### 2.1. Knowledge Graphs

Firstly, a knowledge graph is generally defined as a structured representation of knowledge that consists of entities and relations. In a knowledge graph, entities represent specific objects or concepts, while relations signify the semantic connections between entities [25,26]. Knowledge graphs graphically represent real-world entities and their interrelations, supporting complex queries and inference, thus enhancing the semantic understanding capabilities of deep learning models [27,28]. In this study, the knowledge graph is utilized not only as a data structure to organize and store specialized knowledge related to cucumber diseases, but also to enhance the accuracy and efficiency of disease diagnosis through integration with deep learning models.

In traditional agricultural disease detection research, the isolation of data and fragmentation of information often make it challenging for decision systems to effectively predict and manage diseases [29]. Knowledge graphs provide a novel solution by integrating various types of information resources. In the application of agricultural disease detection, knowledge graphs are not merely aggregations of information; their structure and construction inherently involve complex mathematical principles and logical methods [30,31]. Typically, knowledge graphs consist of entities, relationships, and attributes. Entities correspond to specific objects within the agricultural domain, such as crops, diseases, and meteorological conditions; relationships describe the interactions between these entities, such as “affects” or “treats”; attributes provide detailed information about the entities, such as the type of crop or disease [32,33].

A common method for constructing knowledge graphs involves extracting information from professional literature, agricultural databases, and expert knowledge [34,35]. This process can be facilitated by natural language processing technologies, including entity recognition and relationship extraction. Additionally, knowledge graphs often incorporate machine learning methods to enhance the accuracy and efficiency of disease detection [36]. Specifically, for cucumber disease detection, knowledge graphs can be integrated with the diffusion Transformer model, utilizing the structured knowledge within the graph to optimize the training process of deep learning models. Through this mechanism, the model can focus on information segments most relevant to the current prediction task, such as identifying historical cases and corresponding treatment methods in the graph that are related to the observed symptoms.

In the specific context of cucumber disease detection, the integration of knowledge graphs with the diffusion Transformer allows each analyzed leaf image to consider relevant agricultural knowledge, such as the disease’s lifecycle and the impact of climatic conditions. This not only enhances the model’s interpretability, but also improves the accuracy and practicality of disease diagnosis. Therefore, in this study, knowledge graphs serve not only as carriers of information, but also deeply integrate their mathematical and logical structures into every decision-making process of the model, providing robust technological support for modern agricultural disease management.

### 2.2. Diffusion

Originally utilized in the fields of physics and chemistry to describe the phenomenon of substances achieving a uniform distribution in space through random motion, the diffusion model has been successfully introduced into the field of machine learning in recent years, especially for applications in information propagation, network science, and disease model predictions [37,38,39]. In the domain of agricultural disease detection, the diffusion model is employed to simulate the spread and diffusion of diseases within crops, aiding researchers not only in observing the current state of diseases [40], but also in predicting their future development trends.

In agricultural disease detection, the application of the diffusion model primarily involves simulating the process of disease transmission from one plant to surrounding plants [41]. This process can be described using the following continuous-time random walk model:

(1) $$\frac{dP(t)}{dt}=-\beta LP\left(t\right)$$

where P(t) is a vector representing the probability of disease presence at each node (plant) at time *t*, L is the Laplacian matrix of the graph, and β is the rate of propagation, controlling the speed of disease spread. The model assumes that the probability of disease spreading from one node to its adjacent nodes is directly proportional to the connection strength between these nodes (i.e., the edge weights of the graph). Moreover, considering that the development of diseases in practical applications is influenced not only by local environmental factors, but also by external conditions such as climate and temperature, external influence factors can be integrated into the diffusion model [42], forming a more complex dynamic system model:

(2) $$\frac{dP(t)}{dt}=-\beta LP\left(t\right)+\gamma F\left(t\right)$$

where F(t) represents external influence factors, such as temperature and humidity, and their impact on disease propagation is modulated by the parameter γ. This model allows for more accurate simulation of disease spread within a farm under various external conditions. In the cucumber disease detection methodology, these characteristics of the diffusion model are fully utilized. Firstly, the disease-related knowledge integrated through the knowledge graph provides necessary background information and parameter settings for the diffusion model, such as disease occurrence conditions and influencing factors. Secondly, combined with the powerful data processing capabilities of the Transformer architecture, the model can more effectively learn patterns of disease propagation from a large volume of historical data, enabling the prediction of future disease occurrences and developments.

### 2.3. Transformer

Since achieving significant breakthroughs in the field of Natural Language Processing (NLP) [43], the unique structure and mechanisms of the Transformer model have been extensively applied to various pattern recognition tasks, particularly in image processing and computer vision [44,45]. At its core, the self-attention mechanism allows the Transformer to directly compute dependencies between elements in a sequence, regardless of their positions, thereby effectively capturing key information. In the domain of agricultural disease detection, the Transformer model, through its self-attention mechanism, can more accurately identify and classify disease features in crop images [46,47]. The basic mathematical expression for self-attention is given by:

(3) $$\mathrm{Attention}\left(Q,K,V\right)=\mathrm{softmax}\left(\frac{QK^{T}}{\sqrt{d_{k}}}\right)V$$

where *Q*, *K*, and *V* represent Query, Key, and Value, respectively, which are three matrices obtained from different linear transformations of the input data, and $d_{k}$ is the dimension of the key vectors, a normalization factor used to stabilize the computation results. This mechanism dynamically adjusts the focus of each input element on other elements, with the distribution of weights optimized automatically through the learning process, significantly enhancing the model’s ability to learn the internal structure of the data. In processing image data, the Transformer expands the concept of self-attention through the multi-head attention mechanism, allowing the model to concurrently learn different features of data in various representational subspaces [48]. The expression for multi-head attention is:

(4) $$\mathrm{MultiHead}\left(Q,K,V\right)=\mathrm{Concat}\left({\mathrm{head}}_{1},{\mathrm{head}}_{2},\ldots ,{\mathrm{head}}_{h}\right)W^{O}$$

(5) $${\mathrm{head}}_{i}=\mathrm{Attention}\left(QW_{i}^{Q},KW_{i}^{K},VW_{i}^{V}\right)$$

where $W_{i}^{Q}$, $W_{i}^{K}$, $W_{i}^{V}$, and $W^{O}$ are learnable parameter matrices. This design allows the model to capture information across different representational subspaces, enhancing the dimensionality and complexity of information processing, thereby enabling the model to better distinguish disease features in crop images with complex backgrounds and details [49].

When integrated into cucumber disease detection methods, the Transformer model not only supports image recognition, but also enhances the accuracy and robustness of the detection model by combining structured knowledge extracted from knowledge graphs (such as types of diseases, stages of development, and treatment measures) with image data.

## 3. Materials and Method

### 3.1. Dataset Collection

In this study, a focus was placed on developing a cucumber disease detection system based on a knowledge graph and diffusion Transformer. For the training and validation of the model, a substantial collection of cucumber disease images was required. Through these data, the model is enabled to learn the visual features of various diseases, thereby accurately identifying and classifying different types of cucumber diseases in practical applications. The study covered six major types of cucumber diseases, with the number of images collected for each disease type presented in Table 1.

Cucumber mosaic virus causes mosaic patterns and deformities on cucumber leaves, severely affecting photosynthesis and plant growth. A total of 890 images of this disease were collected. Angular leaf spot is characterized by angular or irregular brownish or dark brown spots on leaves, with approximately 1263 images collected for this disease. Powdery mildew, a common fungal disease on cucumbers, is identified by a white powdery substance on leaf surfaces, with about 1502 images reflecting features of this disease. Anthracnose causes water-soaked, dark brown spots on leaves and fruits, with around 996 images collected. Brown spot leads to round or irregular brown to black spots on leaves, with 1174 images collected in this study. Downy mildew primarily affects leaves, appearing as yellow or white frost-like coverings and can lead to leaf wilting, with 1384 images collected showing characteristics of this disease.

**Figure 1:**
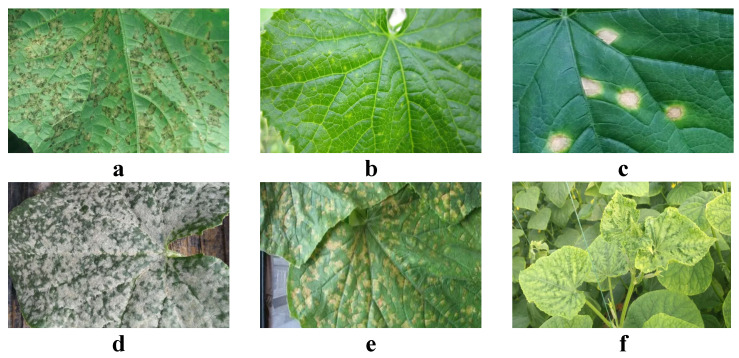
Dataset samples. (**a**) Downy Mildew, (**b**) Brown Spot, (**c**) Anthracnose, (**d**) Powdery Mildew, (**e**) Angular Leaf Spot, and (**f**) Cucumber Mosaic Virus.

All disease images were captured under natural light conditions using high-resolution cameras to ensure sufficient image quality for subsequent image processing and analysis. Images for each disease type were collected at different stages of growth to capture various manifestations of the disease progression. The collected images covered stages from initial symptoms to full-blown disease development, ensuring data diversity and comprehensiveness. Additionally, to minimize the impact of ambient lighting and background, efforts were made to maintain consistent environmental conditions and backgrounds during each photography session, ensuring consistency in image data, as shown in Figure 1.

All collection activities were conducted at multiple agricultural bases in Bayannur, Inner Mongolia Autonomous Region. These bases cultivate a variety of cucumber varieties, representing typical agricultural production conditions of the region. Bayannur was chosen as the collection site because the region’s cucumber cultivation is representative and typical with a rich variety of diseases, making it suitable for such research. Post-collection, the image data underwent preliminary screening to discard images that were out of focus, overexposed, or had severe reflections. For each type of disease, images were annotated by professional plant pathologists to ensure the accuracy of the labels. Annotation details included the type of disease, severity, and affected parts of the plant.

### 3.2. Dataset Preprocessing

#### 3.2.1. Construction of Knowledge Graphs Based on Agricultural Knowledge Corpus

In this study, the process of constructing knowledge graphs serves as the foundation for understanding and analyzing cucumber diseases. Not only do knowledge graphs provide a wealth of background knowledge, they also enhance the interpretability of data and the decision-making capability of models. Initially, key information about cucumber diseases is extracted from a vast array of agricultural texts using NLP techniques, followed by systematic organization using graph database technology. Entity recognition is performed automatically through NLP tools, identifying key terms such as disease names, symptom descriptions, and treatment measures, which become nodes within the knowledge graph. For instance, in the text “Initial yellow spots appear on the lower leaves of the cucumber, gradually spreading to the entire plant”, “yellow spots” and “spreading” are marked as symptom entities. The extraction of relationships involves determining the logical connections between these entities, such as “causes” and “treats”. These relationships define how nodes are connected within the graph, key to understanding disease transmission and treatment methods.

Entity recognition can be accomplished using the Named Entity Recognition (NER) model, typically based on deep learning technologies such as Bi-directional Long Short-Term Memory networks (BiLSTM) combined with Conditional Random Fields (CRF). Mathematically, the NER task can be represented as a sequence labeling problem, aiming to maximize the conditional probability:

(6) $$P\left(y|x\right)=\prod_{i=1}^{n}P\left(y_{i}|x,y_{1}\right)$$

where $x=(x_{1},\ldots ,x_{n})$ is the input text sequence and $y=(y_{1},\ldots ,y_{n})$ is the corresponding label sequence, with $P(y_{i}|x,y_{1})$ representing the conditional probability of the *i*-th label given the input sequence and the previous i−1 labels. Following the extraction of entities and relationships, these are structurally stored using graph database technology to construct a queryable and analyzable knowledge graph. In the graph database, entities and relationships are transformed into nodes and edges. Each node and edge can store additional attributes, such as the severity of the disease and the infection period. Researchers can easily query specific disease information or analyze the interrelationships between diseases using a graph query language.

The structured information in the knowledge graph is used as an additional input to the model, integrated through a specific embedding layer into the diffusion Transformer model, enhancing the model’s ability to recognize images of cucumber diseases, especially in handling complex or rare diseases. The implementation of this method not only improves the efficiency and accuracy of cucumber disease detection, but also advances the development of intelligent agricultural technology, offering new approaches and tools for future disease management in agriculture.

#### 3.2.2. Image Enhancement Methods

**Figure 2:**
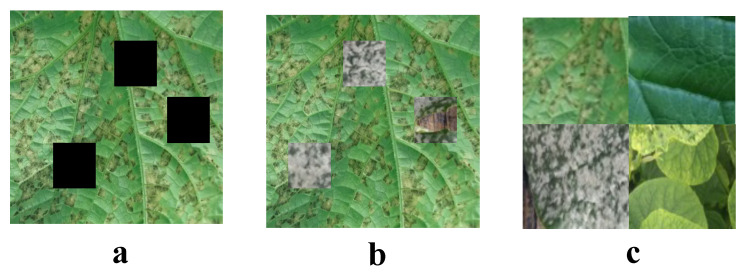
Image enhancement methods. (**a**) Cutout, (**b**) Cutmix, and (**c**) Mosaic.

During the training process of deep learning models, particularly when processing image data, data augmentation techniques are crucial for enhancing model generalizability. A series of data augmentation techniques, including CutOut, CutMix, and Mosaic methods, are employed for processing cucumber disease images, as shown in Figure 2.

The CutOut method involves randomly selecting a small region in an image and setting its pixel values to zero, simulating the occurrence of occlusion. This method is mathematically represented as:

(7) $$I_{cutout}=I⊙\left(1-M\right)$$

where *I* is the original image and *M* is a mask matrix of the same size as the image, with the selected occlusion area marked as 1 and the rest as 0. ⊙ denotes element-wise multiplication. This operation forces the model to focus not only on the occluded part, but also to learn from other features of the image. The CutMix technique involves mixing regions between two images. Specifically, a region is cut from one image and pasted onto the corresponding position in another image, with labels mixed accordingly. The mathematical expression for this is:

(8) $$I_{cutmix}=M⊙I_{A}+\left(1-M\right)⊙I_{B}$$

(9) $$y_{cutmix}=\lambda y_{A}+\left(1-\lambda \right)y_{B}$$

where $I_{A}$ and $I_{B}$ are two images, $y_{A}$ and $y_{B}$ are the corresponding labels, *M* is the chosen binary mask matrix, and λ is the mixing ratio, usually determined by the size of the cut region. This method not only increases image diversity, but also promotes the model’s ability to integrate local and global information. Mosaic data augmentation is achieved by combining randomly cropped regions from four different images to form a new image. This method expands the model’s ability to recognize targets against complex backgrounds:

(10) $$I_{mosaic}=\mathrm{concat}\left(\mathrm{crop}\left(I_{1}\right),\mathrm{crop}\left(I_{2}\right),\mathrm{crop}\left(I_{3}\right),\mathrm{crop}\left(I_{4}\right)\right)$$

where crop(I) represents a randomly cropped region from image *I* and concat denotes the concatenation of these cropped images into a new image.

Through these advanced data augmentation techniques, the model is exposed to more diverse and complex image scenarios during training, thereby enhancing its generalizability. This is particularly important for the cucumber disease detection model, as images of diseases in actual applications are often affected by various factors. By simulating these disturbances, data augmentation techniques ensure the robustness and accuracy of the model when facing different environments and conditions.

### 3.3. Proposed Method

#### 3.3.1. Overall

**Figure 3:**
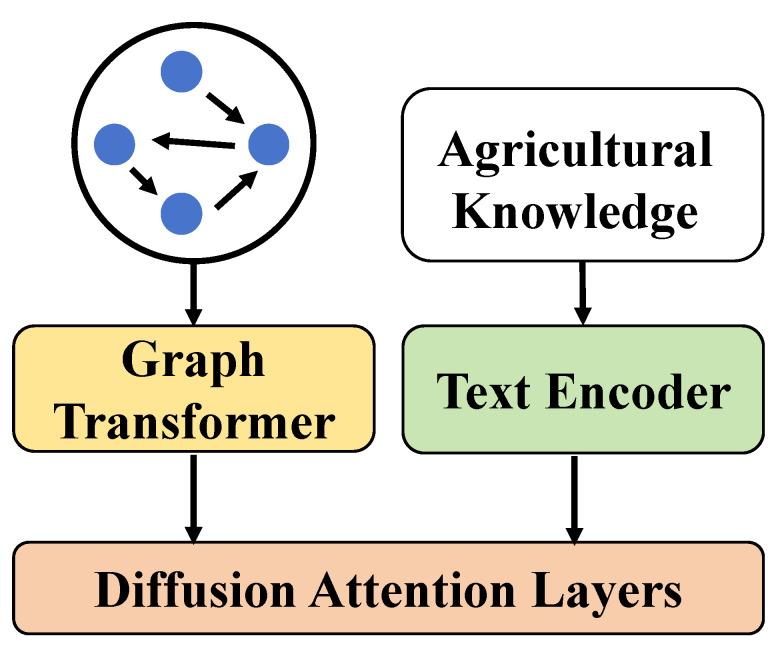
The overall structural workflow of the cucumber disease detection system based on knowledge graph and diffusion Transformer proposed in this paper.

This paper proposes a cucumber disease detection method that integrates a knowledge graph with a diffusion Transformer system. The aim is to enhance the accuracy and efficiency of disease detection through the combination of deep learning and domain knowledge. The design of the system unfolds through several modules: construction of the knowledge graph, design of the diffusion Transformer model, implementation of the diffusion attention mechanism, and the innovative application of the diffusion loss function, as shown in Figure 3.

Initially, the system integrates knowledge of cucumbers and their diseases, including types of diseases, conditions of occurrence, and typical symptoms, through the construction of an agricultural knowledge graph. The construction of the knowledge graph is based on a wealth of agricultural literature and expert knowledge, utilizing natural language processing technologies to extract key information from texts, and structuring this information through entity recognition and relationship extraction techniques to form a network of relationships between entities. This information serves as auxiliary data in model training and prediction, providing background knowledge support. Based on the support of the knowledge graph, the system designs the diffusion Transformer model, a special deep learning model that merges the self-attention mechanism of the Transformer with the propagation characteristics of graph knowledge. In its implementation, the model not only employs standard self-attention layers to process the input image data, but also enhances its capability to recognize disease features through interaction with the knowledge graph. To further increase the model’s sensitivity to disease information, the system introduces the diffusion attention mechanism. This mechanism, building on traditional attention mechanisms, considers the information propagation pathways in the graph structure, enabling the model to focus more on the graph entities and relationships relevant to the current diagnostic task during image data processing. This mechanism adjusts the attention weights, allowing the model to focus more on important features. Finally, to optimize the learning process and enhance the generalization capability of the model, the diffusion loss function is designed. This loss function accounts for the varying difficulties the model may face in classifying diseases, especially for types of diseases that are less frequent or harder to recognize, by dynamically adjusting the loss weights. Through the close integration of these steps, the system not only effectively identifies cucumber diseases, but also offers a new solution for agricultural disease detection by learning a deep integration of graph knowledge and image features.

#### 3.3.2. Implementation of Knowledge Graph-Diffusion Transformer

**Figure 4:**
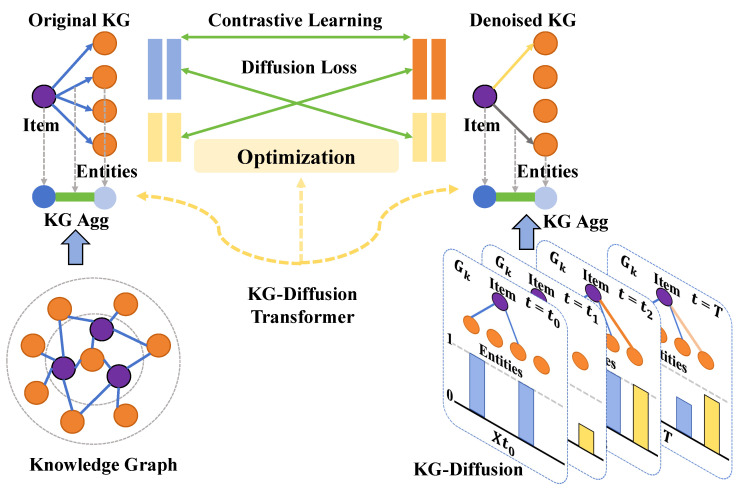
Implementation of knowledge graph-diffusion Transformer.

In this study, a novel knowledge graph-diffusion Transformer model was developed to effectively integrate domain-specific knowledge from agriculture with modern deep learning technology, as shown in Figure 4. This model is designed to delve into the complexities of cucumber disease detection and provide a system for accurately identifying various diseases. The following outlines the specific implementation and design parameters of the model, along with a mathematical analysis and the advantages of this design approach.

The knowledge graph-diffusion Transformer model is an enhanced version of the standard Transformer architecture that incorporates an agricultural knowledge graph as an auxiliary source of information to augment the model’s capability in recognizing cucumber disease images. The core architecture of the model consists of multiple layers of self-attention, each comprising:

1. Self-Attention Mechanism: Each self-attention layer contains multiple heads, with each head processing different representational subspaces of the input data. The standard configuration includes 12 heads, each with a dimensionality of $d_{k}=64$. This design enables parallel processing of the multidimensional features of the input data.
2. Feed-Forward Network: Following the self-attention layer, the data passes through a feed-forward network comprising two linear transformations with a ReLU activation function in between. The first linear layer expands the dimensions to 2048, while the second linear layer compresses them back to a model dimension of 768.
3. Normalization and Residual Connection: The output of each sublayer (self-attention and feed-forward network) is passed through a residual connection followed by layer normalization. This design aids in improving the flow of gradients during training and accelerates model convergence.

To integrate knowledge graph information, an innovative approach was adopted by incorporating the structure of the knowledge graph into the self-attention mechanism. Specifically, the model not only learns features from image data, but also learns the relationships and attributes between entities in the knowledge graph, implemented through the following equation:

(11) $$\mathrm{Knowledge-Enhanced Attention}\left(Q,K,V,E\right)=\mathrm{softmax}\left(\frac{QK^{T}+QE^{T}}{\sqrt{2d_{k}}}\right)V$$

where *E* represents the entity feature matrix extracted from the knowledge graph, which interacts with the query matrix *Q*, thereby incorporating graph information into the model. This design enables the Transformer not only to focus on the image content itself, but also to consider professional agricultural knowledge related to the image content, such as disease relationships and management methods. Through this design, the knowledge graph-diffusion Transformer model offers several advantages in cucumber disease detection.

1. Information integration capability: The model can simultaneously process image data and unstructured text data (extracted from the knowledge graph), providing a more comprehensive decision support through multimodal learning.
2. Context awareness capability: By incorporating the knowledge graph, the model learns not only image features, but also grasps the development process of diseases and related agricultural measures, offering more accurate disease prediction.
3. Generalization performance: The introduced diffusion attention and diffusion loss function help the model generalize better to new and unseen disease types, reducing the risk of overfitting.

This approach not only enhances the accuracy of cucumber disease detection, but also provides new technical directions and application possibilities for other crop disease detection efforts. Future work will continue to optimize the construction of the knowledge graph and the information fusion mechanism, exploring its potential in broader agricultural applications.

#### 3.3.3. Diffusion Attention

**Figure 5:**
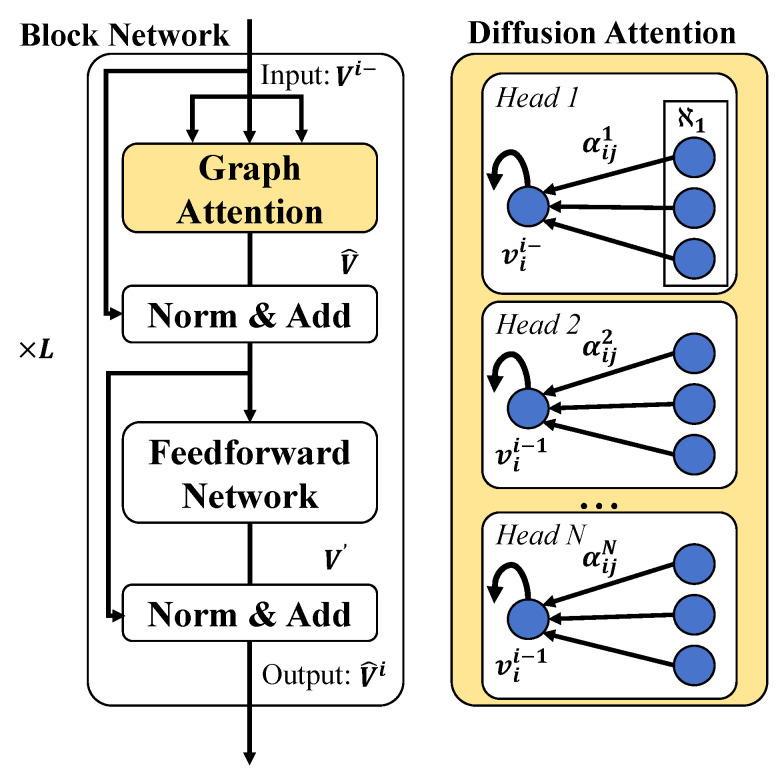
Diffusion attention architecture.

In the field of deep learning, the Transformer model is widely applied to various sequence processing tasks due to its robust self-attention mechanism. However, when dealing with data that exhibit complex relationships and dependencies, such as in agricultural disease detection, the original self-attention mechanism may not fully capture and utilize the deep connections between these data. Therefore, a new attention mechanism called diffusion attention is proposed in this study, aimed at enhancing the predictive performance of the model by integrating structured information from knowledge graphs, as shown in Figure 5.

In the conventional Transformer model, the core formula of the self-attention mechanism is as follows:

(12) $$\mathrm{Attention}\left(Q,K,V\right)=\mathrm{softmax}\left(\frac{QK^{T}}{\sqrt{d_{k}}}\right)V$$

where *Q*, *K*, and *V* represent the Query, Key, and Value matrices, respectively, all of which are derived from different linear transformations of the input data; $d_{k}$ is the dimensionality of the key vectors, used to normalize the computation results to prevent the vanishing gradient problem. This mechanism allows the model to automatically adjust focus while processing data, concentrating on information most relevant to the current task. The diffusion attention mechanism builds upon the traditional self-attention by further incorporating structured information from knowledge graphs, thereby enhancing the model’s capability to comprehend complex data relationships in agricultural disease detection. The central concept is to use the entity relationships in the knowledge graph to guide the distribution of attention, enabling the model to focus more on features that significantly impact the prediction task. The specific mathematical expression is as follows:

(13) $$\mathrm{Diffusion Attention}\left(Q,K,V,A\right)=\mathrm{softmax}\left(\frac{QK^{T}+QA^{T}}{\sqrt{2d_{k}}}\right)V$$

where *A* represents the relationship matrix extracted from the knowledge graph, which interacts with the query matrix *Q*, guiding the distribution of attention. The inclusion of $QA^{T}$ ensures that the model considers not only the traditional content similarity (i.e., $QK^{T}$), but also the strength of relationships between entities, thus offering an attention scoring mechanism that combines content and structural information.

The design of incorporating $QA^{T}$ is based on the following mathematical and practical considerations. From a mathematical perspective, adding $QA^{T}$ to the original $QK^{T}$ means that the model simultaneously considers the direct content similarity between elements and the structural similarity mediated by paths in the knowledge graph. This design allows the attention mechanism to consider more comprehensive information when calculating the importance of each element relative to others, thereby enhancing the accuracy of decisions. From a practical application perspective, in agricultural disease detection, the occurrence and development of many diseases are not only related to the observed symptoms, but also to various factors, such as the type of crop and the planting environment. By integrating this information, diffusion attention enables the model to focus not only on obvious symptoms, but also on the complex connections between these background factors, such as how the same disease may manifest differently in different crops. Moreover, empirical evidence has shown that models incorporating diffusion attention achieve higher accuracy and robustness in disease detection tasks compared to those using traditional self-attention. This is because the model can more effectively utilize all available information, including direct image features and indirect knowledge graph information.

#### 3.3.4. Diffusion Loss Function

In deep learning, the design of the loss function directly impacts the training effectiveness of models. The diffusion loss function proposed in this paper is designed to optimize cucumber disease detection models by incorporating structural information from knowledge graphs, thus better adapting to the complexities of agricultural disease detection tasks. In conventional Transformer models, the commonly used loss function is the cross-entropy loss function, suitable for multi-class classification problems. For a given input *x* and the model’s probability distribution p(y|x), with the true label as *y*, the cross-entropy loss function is defined as:

(14) $$L_{CE}\left(y,p\right)=-\sum_{c=1}^{C}y_{c}\log p_{c}$$

where *C* is the total number of classes, $y_{c}$ is the one-hot encoding of the true label, and $p_{c}$ is the probability distribution predicted by the model. This loss function performs well in typical classification tasks, but has limitations when dealing with complex agricultural disease data, especially in the presence of class imbalance and multi-label conditions. To address the specifics of cucumber disease detection, the diffusion loss function was designed, which not only considers the accuracy of classification, but also integrates structural information from the knowledge graph to enhance the model’s ability to learn complex relationships. The specific formulation of the loss function is as follows:

(15) $$L_{Diffusion}\left(y,p,G\right)=L_{CE}\left(y,p\right)+\lambda L_{Graph}\left(y,p,G\right)$$

where $L_{CE}\left(y,p\right)$ is the basic cross-entropy loss, $L_{Graph}\left(y,p,G\right)$ is the graph loss, and λ is a weighting coefficient used to balance these two parts. The graph loss is defined as:

(16) $$L_{Graph}\left(y,p,G\right)=\sum_{i=1}^{N}\sum_{j\in N(i)}w_{ij}{∥p_{i}-p_{j}∥}^{2}$$

where *N* represents the total number of samples, N(i) denotes the set of samples connected to sample *i* in the knowledge graph, $w_{ij}$ is the weight of the graph connection between samples *i* and *j*, and $p_{i}$ and $p_{j}$ are the predicted probability distributions for these two samples, respectively. This formulation allows the model not only to learn features of individual samples, but also to understand relationships between samples, especially those directly connected in the knowledge graph.

The introduction of $L_{Graph}\left(y,p,G\right)$ enables the diffusion loss function to encourage the model to consider dependencies between samples while learning individual sample features. The advantages of this design include:

1. Enhanced classification performance: In traditional loss functions, each sample is processed independently. Diffusion loss considers relationships between samples, aiding the model in learning more complex patterns, particularly effective in datasets with class imbalance, thereby significantly improving the recognition rate for minority class samples.
2. Improved model generalization: By integrating structural information from the knowledge graph, the diffusion loss function helps the model understand and utilize these structural features, thus making more accurate predictions even when faced with unseen but structurally similar training samples.
3. Multi-label problem resolution: In multi-label disease detection tasks, different diseases may influence each other. Through diffusion loss, the model can better handle such complex relationships, enhancing the accuracy of multi-label classification.

In summary, by combining the knowledge graph and diffusion Transformer, the diffusion loss function proposed in this study effectively addresses the multi-label and imbalance issues in cucumber disease detection while enhancing the model’s understanding of disease dynamics. This provides a new technical means for agricultural disease management. Future work will continue to explore the expansion and deepening of the knowledge graph and the potential applications of this loss function in broader agricultural data processing.

### 3.4. Evaluation Metrics

In the assessment of the cucumber disease detection model, multiple key indicators have been selected to comprehensively evaluate the model’s performance and practicality. These indicators not only reflect the model’s accuracy and efficiency in identifying diseases, but also pertain to the model’s response speed in practical applications. Precision is one of the critical metrics for measuring the correctness of the model’s predictions, reflecting the proportion of images correctly identified as diseased among those recognized by the model. Recall, another important performance metric, measures the proportion of diseased images detected by the model out of all actual diseased images. Accuracy, the most intuitive performance metric, represents the proportion of correct predictions among all predictions, whether diseased or not.

Mean Average Precision (mAP) is a commonly used metric in multi-class or multi-label problems, especially in object detection tasks. It first calculates the Average Precision (AP) for each category and then averages these APs across all categories. Frames Per Second (FPS) measures the speed at which the model processes images, i.e., how many frames per second the model can handle. This is a crucial metric for evaluating the model’s real-time performance, particularly important in applications requiring rapid response. The calculation of FPS is relatively simple, being the reciprocal of the time taken by the model to process a single image. These metrics together form a comprehensive assessment of the model’s overall performance, ensuring that the model not only performs excellently in laboratory tests, but also operates effectively in field applications.

### 3.5. Baseline

In evaluating the effectiveness of the detection method presented in this article, various advanced object detection models are employed as baselines. These baseline models include SSD [50], YOLOv8 [51], YOLOv9 [52], TinySegformer [20], and Detection Transformer (DETR) [53], all of which have demonstrated exceptional performance in multiple image recognition tasks. SSD, a single-stage object detection model, performs object detection across multiple scales of feature maps, achieving high efficiency and accuracy. The core of SSD involves the use of multi-scale feature maps and default boxes (anchor boxes), enabling object detection across various image sizes.

The YOLO series models are well-known in the object detection domain for their speed and accuracy. YOLOv8 and YOLOv9, the latest iterations in the series, predict multiple bounding boxes and class probabilities across the entire image in a single inference for object detection.

TinySegformer, based on the Transformer architecture, is a lightweight model particularly suited for scenarios requiring rapid processing. DETR introduces the Transformer architecture to address problems in object detection, processing objects in images from a global perspective. The core of DETR lies in its end-to-end design, predicting all objects’ classes and bounding boxes directly, eliminating the need for manually designed components typical in traditional detection systems.

By comparing the performance of these baseline models with the method based on the knowledge graph-diffusion Transformer, a comprehensive assessment of the new method’s effectiveness and advantages in cucumber disease detection is achieved. Additionally, the decision to compare the proposed model with traditional object detection models, such as SSD, YOLO series, and DETR, rather than with graph-based models, is grounded in several considerations: primarily, cucumber disease detection is fundamentally an image recognition and object detection task aimed at accurately identifying disease types and locations from images. Comparing with conventional object detection models provides a direct demonstration of the advantages of the proposed model in processing visual information. Moreover, while graph-based models inherently excel at handling structured data, they may face challenges in efficiency and scalability when processing large-scale image data. The proposed model explores a new pathway by integrating structured information from knowledge graphs with the robust image processing capabilities of deep learning, aiming to efficiently utilize the rich semantic information of graph data while maintaining model efficiency.

### 3.6. Experimental Setup

Concerning computational resources, the experiments utilized two NVIDIA GeForce RTX 3090 GPUs (Santa Clara, CA, USA), each equipped with 24 GB of memory. These high-performance GPUs provide sufficient computational power for deep learning models, especially when dealing with large datasets. The large memory capacity of the GPUs allowed for a batch size of 64 in this study. This setting was determined based on the memory capacity of the GPUs and the computational demands of the target model, aiming to balance the efficiency and effectiveness of model training. In terms of storage configuration, the experiments used a 2 TB NVMe SSD (Santa Clara, CA, USA) with a read-write speed of up to 3500 MB/s. The high-speed SSD significantly improves data reading speeds, thus accelerating data loading times during model training, which is crucial for deep learning models. Regarding the hyperparameter settings for model training, the initial learning rate was set at 0.001, with the optimizer being stochastic gradient descent (SGD) with momentum, where the momentum parameter was set to 0.9. To address potential overfitting during training, a learning rate decay strategy and early stopping were implemented. Specifically, the learning rate decay strategy involves multiplying the learning rate by 0.95 after each epoch, gradually reducing the learning rate to stabilize the training process. The early stopping strategy is to cease training when there is no improvement in loss on the validation set for ten consecutive epochs, which helps to preserve the best model and prevent overfitting. Additionally, the dataset in this study was divided into training, validation, and testing sets, with ratios of 70%, 15%, and 15%, respectively. This division ensures the full utilization of data and effectively evaluates the generalization ability of the model.

## 4. Results and Discussion

### 4.1. Disease Detection Experimental Results

This section assesses the performance of various deep learning models on the task of cucumber disease detection through a series of quantitative performance indicators. The purpose of the experiments is to validate the advantages and characteristics of the proposed method in comparison to other existing deep learning models (such as SSD, YOLOv8, YOLOv9, DETR, and TinySegformer) across key performance metrics: precision, recall, accuracy, mAP, and FPS. By comparing the performance of these models, an analysis from both theoretical and practical perspectives is provided, which guides future model optimization and application. The experimental results are displayed in Table 2.

The results from the table indicate superior performance across multiple key indicators for the proposed method. Specifically, the proposed method achieved a precision of 0.93, a recall of 0.89, an accuracy of 0.92, an mAP of 0.91, and an FPS of 57. In contrast, the traditional SSD model exhibited a precision of 0.80, a recall of 0.78, an accuracy of 0.79, an mAP of 0.79, and an FPS of 21; the YOLOv8 model showed a precision of 0.84, a recall of 0.82, an accuracy of 0.83, an mAP of 0.83, and an FPS of 39; YOLOv9 demonstrated a precision of 0.87, a recall of 0.85, an accuracy of 0.86, an mAP of 0.86, and an FPS of 26; DETR had a precision of 0.89, a recall of 0.87, an accuracy of 0.88, an mAP of 0.88, and an FPS of 44; and TinySegformer recorded a precision of 0.91, a recall of 0.89, an accuracy of 0.90, an mAP of 0.90, and an FPS of 43. Theoretical analysis suggests that the performance differences among the models stem from their architectural features and processing mechanisms. SSD, as an earlier single-stage detection model, although fast, exhibits lower precision and recall in handling complex backgrounds and small targets. YOLOv8 and YOLOv9, as improved versions of the YOLO model, show enhancements in both speed and accuracy, particularly YOLOv9, which maintains good detection performance while offering a high FPS. DETR uses a Transformer as its backbone network, eliminating complex predefined anchor points and improving detection accuracy through global optimization and direct boundary box prediction. TinySegformer, a lightweight model based on the Transformer, is particularly suitable for scenarios requiring quick processing. The proposed method, by integrating the knowledge graph and diffusion Transformer, further enhances detection accuracy and efficiency, especially in handling the complex situations and disease characteristics specific to the agricultural field.

The proposed method achieves superior performance because it not only considers the visual features of the images, but also integrates rich background information from the knowledge graph. Using the diffusion attention mechanism effectively utilizes this information to guide the detection process, enhancing the model’s capability to recognize and classify diseases. Additionally, the introduction of the diffusion loss function focuses the training process more on hard-to-recognize or rare disease categories, effectively addressing class imbalance problems and improving the model’s generalization ability. This deep integration of domain knowledge with deep learning technology significantly enhances model performance in practical applications, particularly in terms of precision and frames per second, ensuring the model’s practicality and response speed in operational settings.

### 4.2. Knowledge Graph Ablation Experiment

The purpose of this experiment was to explore the impact of integrating Knowledge Graph (KG) embeddings into various target detection models for detecting diseases in cucumbers. By incorporating KG embeddings into traditional target detection models, the experiment aimed to assess whether this combination could enhance the models’ precision, recall, accuracy, mean Average Precision (mAP), and processing speed (Frames Per Second, FPS). The experimental results comprehensively displayed the performance changes in each model before and after the integration of KG embeddings, thereby validating the contribution of knowledge graph information in enhancing model performance and its applicability in agricultural disease detection.

In Table 3, models such as SSD, YOLOv8, YOLOv9, DETR, and TinySegformer were tested as baselines and compared with their versions integrated with KG embeddings. While baseline models already performed well within the domain of target detection, their integration with knowledge graphs showed varying degrees of performance enhancement. For instance, the SSD model improved its precision from 0.80 to 0.82 and its recall from 0.78 to 0.79 after incorporating KG embeddings, indicating that the addition of semantic information can enhance the model’s ability to recognize disease features. Similarly, the YOLOv8 and DETR models also demonstrated improved performance, suggesting that KG embeddings provided crucial information that aided in enhancing disease detection capabilities.

Theoretically, the integration of KG embeddings provides the model with additional background knowledge and relational information between entities, aiding the model in better understanding and processing complex disease features. This embedding typically manifests as adding extra dimensions to the model’s input layer, which include feature vectors extracted from the knowledge graph. Such feature fusion allows the model to learn and infer not only based on image data, but also using the relational data from the graph, thereby enhancing the model’s ability to handle complex data. Additionally, the structural information from the knowledge graph can help the model improve recognition of rare diseases in cases of uneven sample distribution or sparse data.

However, the results also indicated that while all models showed improved performance after integrating KG embeddings, the degree of improvement was not significant, particularly in the TinySegformer model, where the performance enhancement was negligible. This may suggest that mere feature fusion is insufficient to fully leverage the potential of knowledge graphs, requiring deeper model structural optimizations and algorithm adjustments to better utilize these complex relational data. This may be because simple feature fusion does not alter the intrinsic structure and learning mechanisms of traditional models, which fundamentally lack the capability to process and understand graph-structured data. In contrast, the proposed method in this paper optimizes the model architecture, particularly through the design of Diffusion Attention and Diffusion Loss Function, enabling the model to understand and utilize information from the knowledge graph more effectively, thereby enhancing the identification and classification of complex agricultural diseases.

### 4.3. Diffusion Attention Ablation Experiment

This section presents a comparative study of three different attention mechanisms, i.e., traditional self-attention, multi-head attention, and diffusion attention, to evaluate the performance enhancement of diffusion attention in cucumber disease detection. The experiment is designed to ascertain the effectiveness and efficiency of each attention mechanism in handling complex image data, especially in enhancing the model’s capability to capture features of agricultural diseases. The experimental results are presented in Table 4.

The table shows significant performance differences among the three attention mechanisms in the task of cucumber disease detection. Specifically, the traditional self-attention mechanism achieves a precision of 0.76, recall of 0.73, accuracy of 0.75, mAP of 0.74, and FPS of 37. The multi-head attention mechanism shows improved performance with a precision of 0.85, recall of 0.81, accuracy of 0.83, mAP of 0.84, and FPS of 43. In contrast, the model employing diffusion attention exhibits the best performance across all metrics with a precision of 0.93, recall of 0.89, accuracy of 0.92, mAP of 0.91, and FPS of 57. Theoretically, the self-attention mechanism, by calculating the weight distribution among elements within the input data, autonomously focuses on significant features. However, it does not consider the potential relationships or external information between elements, which may result in under performance in handling complex or noisy image data. The multi-head attention mechanism, by dispersing attention across multiple heads that process different representational subspaces in parallel, enhances the model’s learning capacity and performance to some extent. Yet, this mechanism still relies on internal data features without external guidance.

The superiority of the diffusion attention mechanism lies in its integration of additional information from knowledge graphs, which provides crucial clues about the relationships between data elements, especially in an application scenario like agricultural disease detection, where the associations and complexity between different diseases are considerable. Diffusion attention enhances sensitivity to image features and strengthens the model’s understanding of complex relationships by incorporating structural information from graphs into the attention mechanism. Furthermore, this mechanism, by reinforcing the model’s focus on graph entities and connections relevant to the current task, enables more accurate disease identification and classification, particularly in cases of imbalanced samples or when features are not prominent.

In summary, the introduction of diffusion attention significantly enhances the precision and efficiency of cucumber disease detection. The experiment demonstrates its value in utilizing deep learning technology for processing agricultural disease images. This novel attention mechanism not only improves model performance, but also provides new perspectives and methods for complex data analysis. Future research could further explore how to optimize and expand this mechanism to accommodate a broader range of applications and challenges.

### 4.4. Diffusion Loss Function Ablation Experiment

This section aims to evaluate the performance enhancement of the diffusion loss in cucumber disease detection by comparing three different loss functions: traditional cross-entropy loss, focal loss, and diffusion loss. The purpose of the experiment is to clarify the impact of different loss functions on the training outcomes of models, especially when dealing with complex agricultural disease image data and how these loss functions affect the learning process and the ultimate detection performance of the models. The experimental results are presented in Table 5.

The experimental results indicate significant performance disparities among the three loss functions in the task of cucumber disease detection. The traditional cross-entropy loss function shows relatively lower performance in precision, recall, accuracy, and mAP, achieving 0.74, 0.71, 0.72, and 0.72, respectively, with an FPS of 34. The focal loss shows improved performance with a precision of 0.87, recall of 0.84, accuracy of 0.86, mAP of 0.87, and an FPS of 41. In contrast, the model utilizing diffusion loss outperforms the others on all metrics, achieving a precision of 0.93, recall of 0.89, accuracy of 0.92, mAP of 0.91, and an FPS of 57. Theoretically, the cross-entropy loss function is a fundamental method for loss calculation, mainly suitable for simple multi-class classification tasks. It optimizes the model by calculating the difference in information entropy between the predicted probability distribution and the actual labels. However, it may not be effective in handling data with sample imbalances or complex features. Focal loss, designed to address class imbalance issues, improves recognition rates for minority class samples by adjusting focus away from easy-to-classify examples and increasing penalties for difficult-to-classify ones, thus performing better in more challenging tasks.

The advantage of diffusion loss lies in its consideration of not only the accuracy of individual sample classification, but also the integration of structural information from knowledge graphs. By incorporating relationship weights between samples in the loss calculation, diffusion loss enables the model to consider dependencies between samples while learning individual features. This design is particularly suited to handling data with complex interactions, such as different disease types in agricultural disease detection, and it dynamically adjusts loss weights to enhance the model’s ability to learn complex data relationships. This has been crucial in improving the model’s generalization capability and accuracy.

Overall, by merging domain-specific knowledge with structured data information, diffusion loss significantly enhances the precision and efficiency of cucumber disease detection. The experiment demonstrates its substantial advantages in enhancing model performance, especially in situations with high class imbalance and data complexity. This novel loss function not only improves detection accuracy, but also provides new methods and perspectives for handling complex agricultural data, offering robust support for the technological development of agricultural disease management. Future research could further explore how to optimize and expand this loss function to accommodate a broader range of applications and challenges.

### 4.5. Model Lightweight Deployment

**Figure 6:**
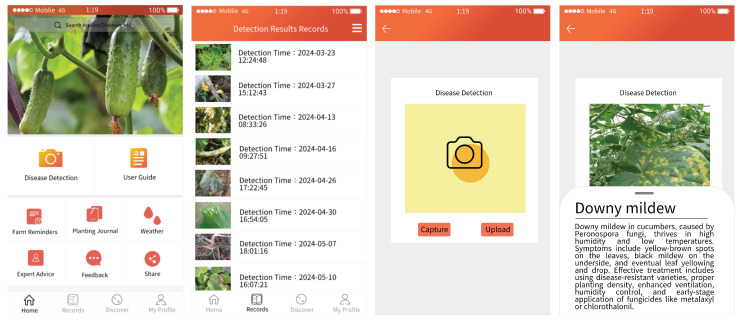
Application screenshot based on our method.

In the development of the cucumber disease detection system, the requirements for mobile application deployment were thoroughly considered, particularly in terms of model lightweighting and deployment, to ensure the system’s efficiency and accuracy on mobile devices such as the Huawei Mate 60, as shown in Figure 6.

This section details the development process of the lightweight model and the key technologies employed. A primary objective of model lightweighting is to reduce the consumption of computational resources and storage space while maintaining model performance. Various lightweighting techniques, including parameter pruning, quantization, and knowledge distillation, were applied. These techniques collectively reduced the model’s parameter count and computational complexity, making it suitable for mobile hardware environments. Parameter pruning, a common technique for model reduction, was utilized to decrease the model’s size and enhance inference speed by removing redundant parameters within the neural network. Unstructured pruning was applied, which involves evaluating the importance of parameters to decide on their retention. The importance of a parameter is calculated using the following equation:

(17) $$I\left(w\right)=\sum_{i=1}^{N}\left|w_{i}\cdot \nabla L\left(w_{i}\right)\right|$$

where I(w) represents the importance of parameter *w*, $w_{i}$ represents the weight parameters, $\nabla L(w_{i})$ denotes the gradient associated with the weight, and *N* is the total number of parameters. High importance suggests a significant impact on model performance, indicating that the parameter should be preserved. Quantization reduces the bit representation of parameters to decrease storage and computational demands. Quantization-Aware Training (QAT) was employed, which simulates the effects of quantization during the training process to adapt the model to potential information loss. The quantization operation is defined as:

(18) $$Q\left(v\right)=\mathrm{round}\left(\frac{v}{s}\right)\cdot s$$

where *v* is the original parameter value and *s* is the scale factor for quantization, with round(·) representing the rounding operation. This method transforms model weights from floating-point to fixed-point representations, significantly reducing memory usage. Knowledge distillation is another technique used to improve efficiency, involving a smaller model (the student) learning from the output of a larger model (the teacher) to replicate its performance. During knowledge distillation, the learning objectives for the student model are guided by the following loss function:

(19) $$L=\alpha \cdot L_{CE}\left(y,p_{s}\right)+\left(1-\alpha \right)\cdot T^{2}\cdot L_{KL}\left(p_{t},p_{s}\right)$$

where $L_{CE}$ represents the cross-entropy loss for real label learning by the student model; $L_{KL}$ is the *KL* divergence loss for emulating the teacher model’s soft labels; $p_{s}$ and $p_{t}$ are the predictive results from the student and teacher models, respectively; α is the weight coefficient for the loss terms; and *T* is a temperature parameter that adjusts the smoothness of soft labels.

The application of these lightweighting technologies not only achieved efficient operation on devices like the Huawei Mate 60 (Shenzhen, China), but also significantly reduced resource consumption while maintaining model performance. This deployment provides an effective and feasible technological solution for on-site agricultural disease detection, promising significant practical utility. Future work will continue to explore additional lightweighting technologies and optimization strategies to further enhance model performance and adaptability on mobile platforms.

### 4.6. Limitations and Future Work

This paper proposes an innovative cucumber disease detection system based on the knowledge graph-diffusion Transformer, which not only integrates professional knowledge from the agricultural field, but also incorporates the latest diffusion models and Transformer networks, significantly enhancing the accuracy and efficiency of disease detection.

Although the cucumber disease detection system developed in this study based on the knowledge graph-diffusion Transformer has demonstrated commendable performance, there remain limitations and directions for future research. First, despite the effectiveness of the diffusion attention mechanism in integrating structured information from knowledge graphs to enhance model prediction accuracy, its computational complexity is relatively high, demanding considerable computing resources. This aspect somewhat limits the application of the model on resource-constrained mobile devices. While model lightweighting techniques have been optimized to enable operation on mobile devices, performance and efficiency might still be impacted under extremely resource-limited conditions. Moreover, while the current model has been optimized with the diffusion loss function to handle extremely imbalanced datasets, it still shows limitations in detecting some rare diseases. This is due to the model’s insufficient learning from very few samples of certain diseases, which might lead to suboptimal detection performance. Additionally, the knowledge graph used in this study is static, relying on initial construction and not updated dynamically during model training and usage. As agricultural data continuously accumulates and updates, the static graph may not timely reflect the latest knowledge and information, potentially limiting the model’s capability to address newly emerging diseases or variants.

Addressing these issues, future work could take several directions. Firstly, further exploration and optimization of lightweighting techniques are needed, particularly to reduce resource consumption while maintaining model performance, making it more suitable for deployment on various mobile devices. Secondly, the development of mechanisms for the construction and dynamic updating of knowledge graphs could allow the model to utilize the latest agricultural knowledge and data, enhancing its adaptability and robustness. Additionally, in view of the data imbalance issue, more advanced data augmentation and learning strategies, such as Synthetic Minority Over-sampling Technique (SMOTE) [54] or Generative Adversarial Networks (GAN) [55], could be explored to increase the samples of rare disease classes, thereby improving the model’s performance in disease detection. Through these efforts, it is hoped that the model’s practicality and universality can be further enhanced, better serving the needs of modern agriculture, especially in resource-limited agricultural production environments. Future research may continue to explore more lightweighting techniques and optimization strategies to further improve model performance and adaptability for broader application scenarios and challenges.

## 5. Conclusions

In the current research, an innovative system based on knowledge graph and diffusion Transformer has been developed for the detection of cucumber diseases. This research describes three main innovations. Firstly, the introduction of the diffusion attention mechanism, which significantly enhances the model’s ability to capture complex disease features by integrating structured information from the knowledge graph. Secondly, the use of the diffusion loss function, a novel loss function that effectively addresses the issue of sample imbalance, enhancing the model’s performance in detecting rare diseases. Lastly, the implementation of model lightweight deployment, enabling the system to operate efficiently on mobile devices to meet the needs of rapid on-site diagnosis. Experimental results demonstrate that the proposed method excels in key performance indicators such as precision, recall, and accuracy. Specifically, the method achieved a precision of 93%, a recall of 89%, an accuracy of 92%, and an mAP of 91%, with an FPS rate of 57, significantly higher than other compared models such as SSD, YOLOv8, YOLOv9, DETR, and TinySegformer. Furthermore, the application of diffusion attention and diffusion loss function not only improved the model’s performance, but also enhanced its capability to process complex agricultural data. 

## Figures and Tables

**Table 1:** Number of images per disease type.

Disease Type	Number of Images
Cucumber Mosaic Virus	890
Angular Leaf Spot	1263
Powdery Mildew	1502
Anthracnose	996
Brown Spot	1174
Downy Mildew	1384

**Table 2:** Comparison of cucumber disease detection results.

Model	Precision	Recall	Accuracy	mAP	FPS
SSD [15]	0.80	0.78	0.79	0.79	21
YOLOv8 [16]	0.84	0.82	0.83	0.83	39
YOLOv9 [17]	0.87	0.85	0.86	0.86	26
DETR [18]	0.89	0.87	0.88	0.88	44
TinySegformer [20]	0.91	0.89	0.90	0.90	43
Proposed Method	0.93	0.89	0.92	0.91	57

**Table 3:** Knowledge graph ablation experiment.

Model	Precision	Recall	Accuracy	mAP	FPS
SSD (baseline) [15]	0.80	0.78	0.79	0.79	21
SSD + KG Embeddings [15]	0.82	0.79	0.80	0.80	21
YOLOv8 (baseline) [16]	0.84	0.82	0.83	0.83	39
YOLOv8 + KG Embeddings [16]	0.85	0.84	0.84	0.84	37
YOLOv9 (baseline) [17]	0.87	0.85	0.86	0.86	26
YOLOv9 + KG Embeddings [17]	0.87	0.86	0.86	0.86	25
DETR (baseline) [18]	0.89	0.87	0.88	0.88	44
DETR + KG Embeddings [18]	0.90	0.89	0.89	0.89	40
TinySegformer [20] (baseline)	0.91	0.89	0.90	0.90	43
TinySegformer [20] + KG Embeddings	0.91	0.89	0.90	0.90	43
Proposed Method	0.93	0.89	0.92	0.91	57

**Table 4:** Different attention ablation experiment.

Model	Precision	Recall	Accuracy	mAP	FPS
Self-Attention	0.76	0.73	0.75	0.74	37
Multi-Head Attention	0.85	0.81	0.83	0.84	43
Diffusion Attention	0.93	0.89	0.92	0.91	57

**Table 5:** Different loss function ablation experiments.

Model	Precision	Recall	Accuracy	mAP	FPS
Cross-Entropy Loss	0.74	0.71	0.72	0.72	34
Focal Loss	0.87	0.84	0.86	0.87	41
Diffusion Loss	0.93	0.89	0.92	0.91	57

## Data Availability

The original contributions presented in the study are included in the article, further inquiries can be directed to the corresponding author.
